# Neutrophil-to-high-density lipoprotein cholesterol ratio (NHR) mediates the relationship between abdominal fat index and depression in a cross-sectional study

**DOI:** 10.1186/s12888-025-07498-5

**Published:** 2025-10-29

**Authors:** Qiaohe Wang, Qiulin Luo, Zhuo Tian

**Affiliations:** Department of Special Medicine, Chongqing General Hospital, Chongqing University, No.118 Avenue of Stars, Yubei District, Chongqing, 401147 China

**Keywords:** Subcutaneous adipose tissue, Visceral adipose tissue, Neutrophil-to-high-density lipoprotein cholesterol ratio, Depression, NHANES

## Abstract

**Objective:**

Investigate the link between abdominal adipose tissue index and depression, and assess the mediating effect of the neutrophil-to-high-density lipoprotein cholesterol ratio (NHR) in this association.

**Methods:**

Participants were chosen using datasets from the National Health and Nutrition Examination Survey (NHANES). To assess the associations between abdominal adipose tissue index and depression, a weighted logistic regression analysis was conducted. Adjusted logistic regression models and restricted cubic splines were used to examine the relationship between subcutaneous and visceral adipose tissue index and depression, with subgroup analyses conducted according to demographic characteristics, personal habits, and comorbidities. The mediation model assessed the potential role of NHR as a mediator in the relationship between abdominal adipose tissue index and depression. Depression was defined using the PHQ-9 screening questionnaire (cut-off ≥ 10), this reflects a screening result and not a clinical diagnosis.

**Results:**

The weighted prevalence of depression was determined to be 8.10%. The average participant age was 38.81 years, with females making up 47.86% of the sample and showing a higher prevalence of depression than males. The three weighted binary logistic regression models revealed significant positive correlations between Subcutaneous Adipose Tissue Index (SATI), Visceral Adipose Tissue Index (VATI), and depression. Restricted cubic splines (RCS) analysis showed a linear relationship between VATI and depression (P-overall<0.001, P-nonlinear>0.05), whereas SATI exhibited a nonlinear relationship with depression (P-overall<0.001, P-nonlinear<0.05). Mediation analyses indicated that the associations between SATI, VATI, and depression were partially mediated by NHR.

**Conclusions:**

This study elucidates the associations between SATI, VATI and depression, proposing that these relationships may be partially mediated by NHR. These findings offer a novel perspective on the interplay between fat distribution and mental health, and suggest potential biological markers for future preventive and interventional strategies aimed at addressing depression.

**Supplementary Information:**

The online version contains supplementary material available at 10.1186/s12888-025-07498-5.

## Background

Depression is a prevalent mental health disorder globally, exerting a profound impact not only on individuals’ emotional and psychological well-being but also precipitating a spectrum of physical health complications [1, 2]. The principal symptoms of depression encompass persistent low mood, anhedonia, and sleep disturbances. Importantly, the risk of suicide is markedly elevated among those suffering from depression, with severe cases potentially culminating in suicide, a phenomenon particularly pronounced in the elderly demographic [3]. Furthermore, individuals with depression frequently exhibit cognitive impairments, social withdrawal, and somatic symptoms (such as fatigue and alterations in appetite), all of which collectively contribute to a significant deterioration in quality of life [4, 5]. Depression often coexists with other chronic conditions, such as diabetes [6] and obesity [7], thereby complicating patients’ overall health status.

Obesity, especially visceral fat accumulation, is closely associated with an increased risk of depression [8, 9]. The bidirectional relationship between depression and obesity is especially significant: obesity may heighten the risk of depression through mechanisms involving metabolic disorders and chronic inflammation [10], while individuals with depression may experience weight gain due to behavioral changes (such as a sedentary lifestyle and emotional eating) and neuroendocrine dysregulation [7]. Abdominal adipose tissue serves as both an energy reserve and an endocrine organ, releasing pro-inflammatory cytokines (e.g., interleukin-6, tumor necrosis factor-alpha) and adipokines (e.g., leptin) that can affect central nervous system function by crossing the blood-brain barrier [11]. Research shows that visceral fat accumulation is linked to elevated central inflammatory markers like C-reactive protein, with chronic low-grade inflammation being a key pathological mechanism in depression. Obesity-related metabolic issues and chronic inflammation may increase the risk of depression by affecting the neuroendocrine system, including the hypothalamic-pituitary-adrenal axis, and neurotransmitter functions such as dopamine and serotonin [12, 13]. For instance, the concentrations of 70 proteins within the extracellular vesicles in the brains of individuals diagnosed with depression exhibit abnormalities, with 48 of these proteins being associated with synaptic function and neurotransmitter transport. This observation implies that synaptic damage mediated by inflammation constitutes a fundamental mechanism underlying depression [14]. Insulin resistance associated with obesity and impaired high-density lipoprotein cholesterol (HDL-C) can intensify neutrophil activation, triggering a ‘metabolism-inflammation-neurotoxicity’ cascade [13].

The Neutrophil-to-High-Density Lipoprotein Cholesterol Ratio (NHR) serves as a novel biomarker, integrating inflammatory and metabolic conditions to indicate the balance between pro-inflammatory (neutrophil activation) and anti-inflammatory (HDL-C function) processes [15, 16]. Studies have shown a significant link between high NHR levels and cardiovascular diseases like acute coronary syndrome (ACS), with its role as an independent risk factor confirmed in diabetic patients with ACS [17]. NHR is significantly linked to coronary artery stenosis and independently predicts severe cases [18]. A study on US adults found a positive correlation between high NHR levels and increased depression risk [19], indicating that NHR could be a biomarker for depression risk assessment, which is crucial for early detection and personalized treatment strategies.

In summary, obesity and increased visceral fat are significantly associated with depression. However, current studies frequently use indirect measures like body mass index (BMI) or waist circumference to evaluate abdominal adipose tissue, which may not accurately reflect the distribution of visceral and subcutaneous fat. This research utilizes NHANES data and employs Dual-Energy X-ray Absorptiometry (DXA) for direct measurement of abdominal adipose tissue, with height adjustment for accuracy, offering precise and reliable insights into fat distribution. This methodological approach facilitates a more accurate and systematic examination of the relationship between subcutaneous and visceral fat and depression, while also assessing whether NHR mediates the relationship. Our objective is to explore the specific pathways through which abdominal fat influences depression via inflammation-metabolism pathways, thereby offering a theoretical foundation for the development of early intervention strategies based on NHR, such as anti-inflammatory treatments or metabolic regulation.

## Methods

### Data source and study population

**Fig. 1:**
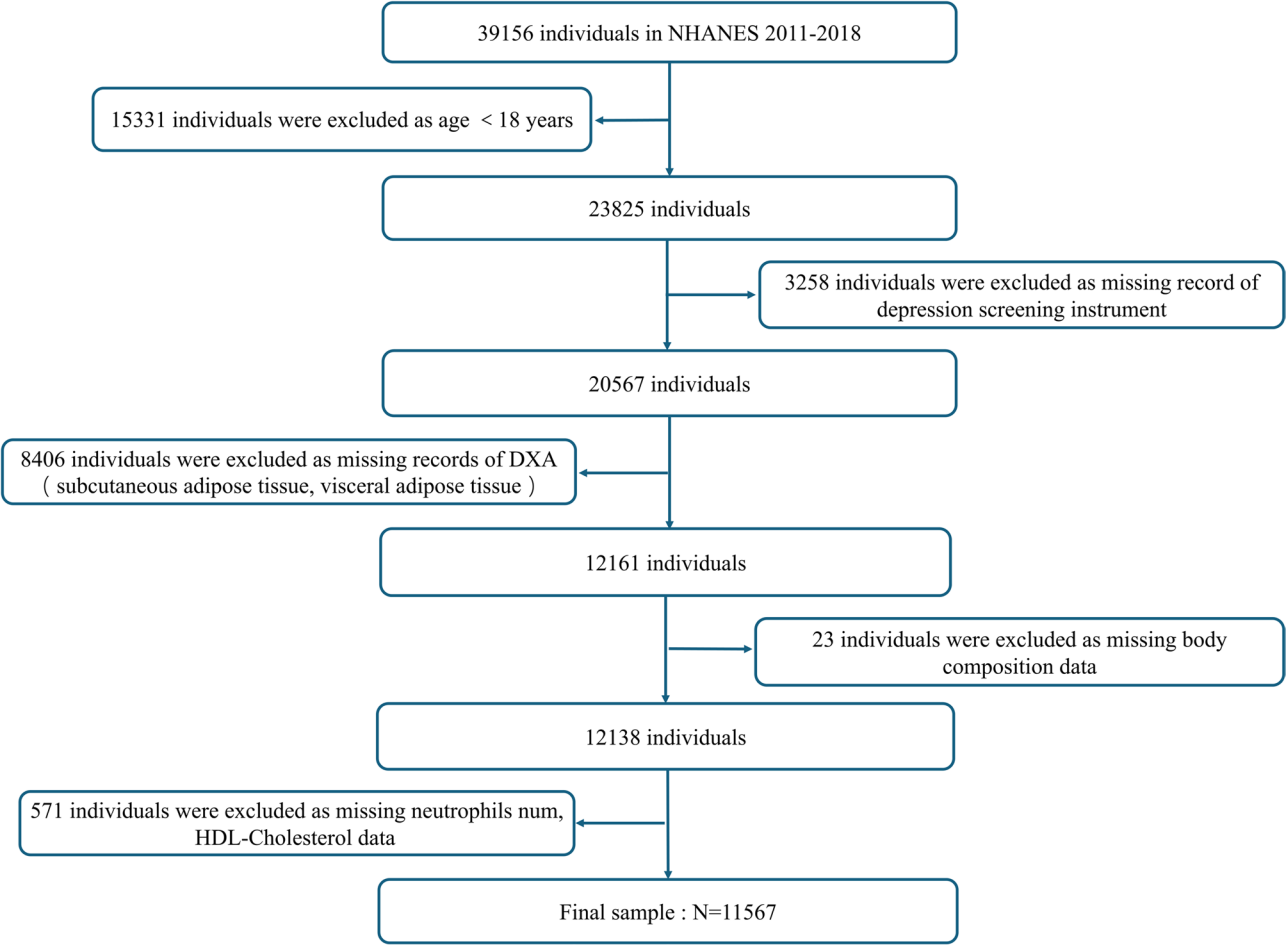
Diagram showing the participants included and excluded from the study

The cross-sectional study utilized datasets from the NHANES initiative, which is a national project aimed at assessing health and nutrition in the United States. Researchers globally had easy access to data via the CDC website. This study utilized subsample data from NHANES spanning 2011 to 2018. The inclusion criteria included: (1) Participants who had full data on depression symptoms were evaluated using the nine-item Patient Health Questionnaire; (2) Patients underwent body composition analysis using Dual-energy X-ray Absorptiometry (DXA) and a physical assessment. Total 39,156 participants were selected. Among them, individuals were excluded for being aged < 18 years(*N* = 15,331), missing record of depression screening instrument(*N* = 3,258), incomplete DXA data(*N* = 8,406), as missing body composition data(*N* = 23), missing neutrophils num and HDL-Cholesterol data(*N* = 571), respectively. Consequently, our final sample included 11,567 individuals (Figure.1).

### Exposure -body composition

Data on body measurements and composition were collected at the Mobile Examination Center (MEC). Participants’ subcutaneous adipose tissue, visceral adipose tissue were obtained using DXA. Subcutaneous Adipose Tissue Index (SATI) was determined as subcutaneous fat mass (kg)/height(m^2^), Visceral Adipose Tissue Index (VATI) was determined as visceral adipose tissue mass(kg)/height(m^2^).

### Outcome –depression

Depression evaluation was conducted with the PHQ-9, a screening tool consisting of nine items that inquire about how often depressive symptoms have occurred over the previous two weeks. The nine-item measure offers response options of ‘not at all’, ‘several days’, ‘more than half the days’, and ‘nearly every day’, with corresponding scores from 0 to 3 [20]. Scores of 10 or higher were considered indicative of clinically relevant depression(CRD), in accordance with the DSM-IV guidelines [19, 21]. This cutoff score has been validated in multiple studies, demonstrating a sensitivity of 88% and a specificity of 88% for major depression [22].

### Mediating factors

The Neutrophil-to-High-Density Lipoprotein Cholesterol Ratio (NHR) was determined by dividing the neutrophil count the ratio of the neutrophil count(×10^3^cells/µL) by the level of high-density lipoprotein cholesterol(HDL-C)(mmol/L) [23]. The neutrophil count and the high-density lipoprotein cholesterol (HDL-C) value were both acquired via laboratory testing.

### Other covariates

Herein, categorical variables included sex (male or female), race [Mexican-American, Other Hispanic, Non-Hispanic White, Non-Hispanic Black, and Other Race (including Multi-Racial)], Poverty-to-Income Ratio (PIR) (≤ 1.3, 1.3–3.5, and >3.5) [24], educational level (less than 9th grade, 9-11th grade, high school or equivalent, some college or AA degree, and college graduate or above), smoking status (never, former smoker, or current smoker), alcohol drinking status (never, mild and moderate, or heavy), High Blood Pressure (HBP) presence (no or yes), diabetes status (no, yes, or prediabetes), physical activity levels (inactive, less active, or active). NHANES questionnaires provided insights into demographic characteristics, personal habits, and comorbidities, while height and weight were measured with a stadiometer and digital scale, and body-composition variables (visceral and subcutaneous adipose tissue) were derived from whole-body DXA scans in the MEC.

### Statistical analyses

Statistical analyses were conducted using EmpowerStats 2.0 (http://www.empowerstats.com, X&Y Solutions, Inc., CA, USA) and Stata 18.0 (StataCorp, College Station, TX, USA). Continuous variables were represented as mean ± standard deviation (SD). Inter-group comparisons employed a weighted logistic regression model. Weighting procedures followed the NHANES Analytic Guidelines. Categorical variables were represented as frequencies and percentages, with comparisons made using the Chi-square test. Weighted logistic regression analysis was used to assess the independent associations of SATI and VATI with depression, with results deemed statistically significant if *P* < 0.05. To investigate potential non-linear relationships, restricted cubic spline (RCS) models were developed for each index. Four knots, corresponding to the 25th, 50th, 75th, and 95th percentiles, were defined using the mkspline function. The spline terms were incorporated into the weighted logistic model, and non-linearity was assessed using the testparm procedure (*P* < 0.05). Odds ratios (ORs) and 95% confidence intervals (CIs) across the spectrum of SATI and VATI were subsequently estimated using xblc and depicted as dose-response curves. Three models were developed to address potential demographic and clinical confounders: Model 1 (unadjusted), Model 2 (adjusted for age, sex, race, and education level), and Model 3 (further adjusted for PIR, smoking status, alcohol consumption, hypertension, physical activity, and diabetes). Potential interactions of SATI and VATI with the aforementioned subgroup variables were analyzed through interaction tests in the final model. In the stratified analyses, SATI and VATI were independently evaluated across 28 distinct strata delineated by nine covariates, resulting in a total of 28 tests. A Bonferroni-corrected significance threshold of *P* < 0.0018 was employed. For the interaction terms (SATI × covariate and VATI × covariate), nine interaction tests were conducted, with the Bonferroni-corrected threshold established at *P* < 0.0056 (0.05/9). The potential mediating effect of NHR on the relationship between SATI, VATI, and depression was assessed using mediator analysis with Stata’s sgmediation package in the SEM framework. To ensure accuracy, 1000 Bootstrap replications were conducted, and the mediation effect’s significance was determined by 95% confidence intervals, considering it significant if zero was not included. The parallel mediation model incorporates individual indicators as mediators. The direct effect is the effect of SATI, VATI on depression without mediators. Indirect effects are the consequences of NHR on depression that were mediated by mediators. The fraction of mediators was estimated by dividing indirect effects by total effect.

## Results

### Participants’ characteristics

The study included 11,567 adult participants, divided into two groups: those with depression (*N* = 979) and those without depression (*N* = 10,588). Table 1 presented the participants’ fundamental demographic, examination, and laboratory data. The weighted prevalence of depression was determined to be 8.10%. The average participant age was 38.81 years, with females making up 47.86% of the sample and showing a higher prevalence of depression than males. Additionally, all baseline characteristics, with the exception of age, demonstrated statistically significant differences (*P* < 0.05). Individuals with depression were more likely to exhibit higher SATI, VATI, and NHR.

**Table 1:** Weighted baseline characteristics associated with depression. Depression status is categorized based on the PHQ-9 screening criteria, with scores of 10 or higher indicating clinically relevant depression (CRD). However, these scores do not constitute a formal clinical diagnosis of depression

	Total	Non-depression	Depression	*P* value
	*n* = 11,567	*n* = 10,588	*n* = 979	
Gender				0.001
Male	52.14(50.91–53.36)	53.26(51.97–54.54)	39.46(35.49–43.58)	
Female	47.86(46.64–49.09)	46.74(45.46–48.03)	60.54(56.42–64.51)	
Race				0.002
Mexican American	10.19(9.67–10.73)	10.45(9.91–11.03)	7.16(5.8–8.82)	
Other Hispanic	7.06(6.63–7.52)	6.88(6.43–7.36)	9.12(7.55–10.98)	
Non-Hispanic White	62.28(61.23–63.33)	62.31(61.21–63.4)	61.94(58.24–65.5)	
Non-Hispanic Black	11.28(10.8–11.79.8.79)	11.26(10.76–11.79)	11.52(9.88–13.39)	
Other Race	9.18(8.66–9.74)	9.09(8.56–9.65)	10.26(8.05–12.99)	
Education level				< 0.001
Less than 9th grade	6.95(6.48–7.46)	6.68(6.2–7.2)	10.01(8.12–12.28)	
9-11th grade	15.71(14.85–16.62)	15.35(14.45–16.29)	19.82(16.86–23.15)	
High school graduate/GED or equivalent	40.97(39.74–42.21)	41.02(39.73–42.32)	40.39(36.32–44.6)	
Some college or AA degree	16.32(15.47–17.22)	16.15(15.26–17.08)	18.33(15.45–21.6)	
College graduate or above	15.62(14.74–16.54)	16.39(15.46–17.38)	6.87(5.01–9.36)	
No recorded	4.42(4.05–4.82)	4.41(4.03–4.82)	4.58(3.33–6.27)	
PIR				< 0.001
≤1.3	23.27(22.39–24.17)	21.74(20.85–22.65)	40.64(36.86–44.54)	
>1.3, ≤ 3.5	31.3(30.2–32.43.2.43)	31.33(30.17–32.51)	31.03(27.31–35.01)	
>3.5	39.04(37.78–40.32)	40.55(39.23–41.88)	21.95(18.3–26.11.3.11)	
No recorded	6.38(5.86–6.96)	6.38(5.83–6.98)	6.38(4.67–8.65)	
Smoking status				< 0.001
Never smoking	58.51(57.29–59.73)	60.4(59.12–61.66)	37.15(33.31–41.17)	
Former smoking	19.4(18.37–20.47)	19.47(18.39–20.6)	18.55(15.47–22.07)	
Current smoking	20.88(19.94–21.86)	18.92(17.97–19.9)	43.19(39.18–47.29)	
No recorded	1.2(1.01–1.43)	1.21(1.01–1.45)	1.11(0.59–2.08)	
Physical Activity				< 0.001
Inactive	20.92(19.99–21.88)	19.77(18.82–20.75)	33.97(30.2–37.95.2.95)	
Less active	12.88(12.07–13.73)	12.77(11.93–13.66)	14.12(11.41–17.36)	
Active	65.98(64.83–67.11)	67.27(66.08–68.44)	51.29(47.19–55.37)	
No recorded	0.22(0.14–0.35)	0.19(0.11–0.31)	0.62(0.22–1.78)	
Alcohol drinking				< 0.001
Never	10.51(9.87–11.18)	10.68(10.01–11.39)	8.57(6.63–11.01)	
Mild and moderate	30.55(29.39–31.73)	31.5(30.28–32.75)	19.73(16.61–23.28)	
Heavy	51.38(50.15–52.61)	50.42(49.13–51.71)	62.3(58.28–66.17)	
No recorded	7.56(6.97–8.19)	7.4(6.79–8.05)	9.4(7.37–11.91)	
HBP				< 0.001
No	73.23(72.12–74.32)	74.2(73.05–75.32)	62.26(58.17–66.17)	
Yes	26.77(25.68–27.88)	25.8(24.68–26.95)	37.74(33.83–41.83)	
Diabetes				< 0.001
No	91.6(90.93–92.22)	91.93(91.23–92.57)	87.9(85.29–90.1)	
Yes	8.37(7.75–9.04)	8.05(7.41–8.74)	12.05(9.85–14.66)	
No recorded	0.02(0.01–0.06)	0.02(0.01–0.06)	0.05(0.01–0.34)	
**BMI (kg/m^2)**	28.91 ± 6.81	28.78 ± 6.70	30.34 ± 7.78	< 0.001
**Age (years)**	38.81 ± 12.31	38.75 ± 12.29	39.54 ± 12.49	0.059
**SATI (kg/m^2)**	0.58 ± 0.31	0.57 ± 0.31	0.68 ± 0.36	< 0.001
**VATI (kg/m^2)**	0.18 ± 0.10	0.17 ± 0.10	0.20 ± 0.12	< 0.001
**NHR**	3.48 ± 1.84	3.44 ± 1.82	3.91 ± 2.05	< 0.001

P value was based on χ2 or analysis of variance test where appropriate

*PIR* Poverty-to-income ratio, *HBP* High blood pressure, *BMI* Body mass index, *SATI* Subcutaneous adipose tissue index, *VATI* Visceral adipose tissue index, *NHR* Neutrophil-to-high-density lipoprotein cholesterol ratio

### Association between SATI, VATI and depression

**Fig. 2:**
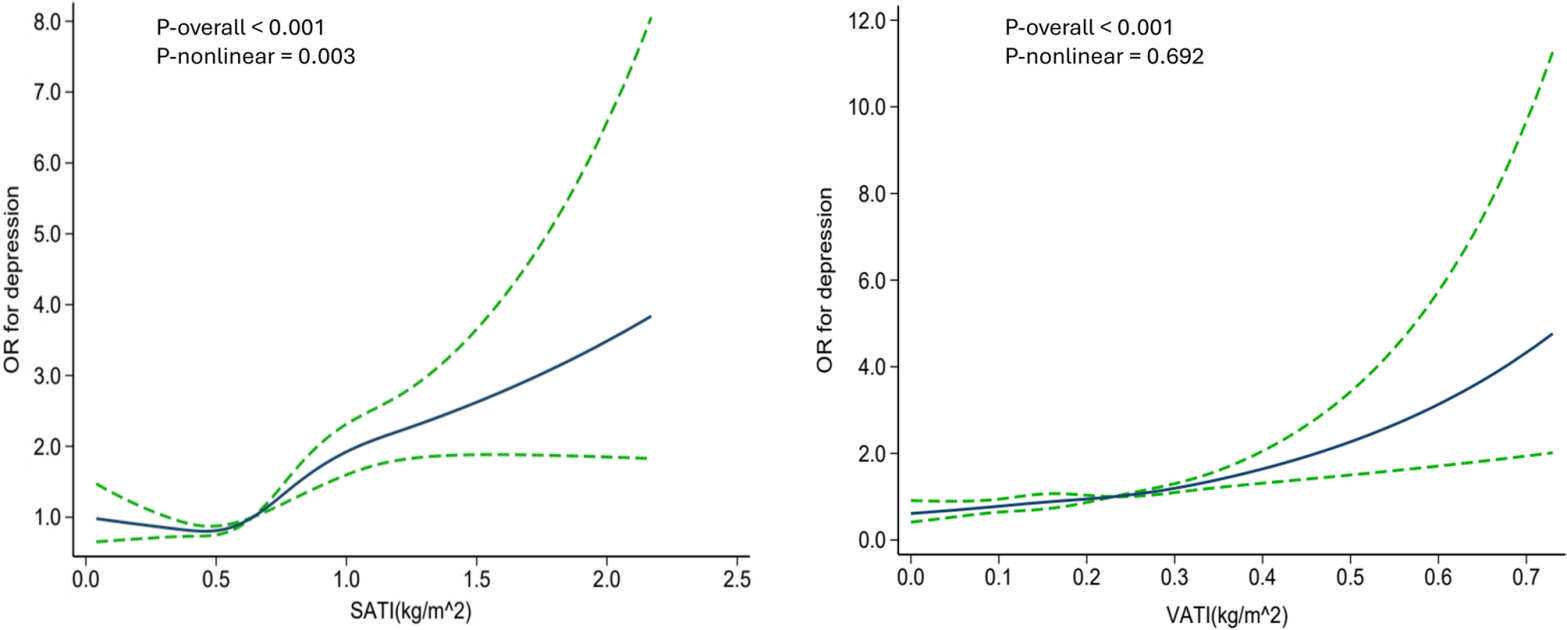
The restricted cubic spline (RCS) was utilized to assess the Subcutaneous Adipose Tissue Index (SATI), Visceral Adipose Tissue Index (VATI), and depression, with the blue lines representing the 95% confidence interval. A key change point was identified at SATI = 0.55 kg/m^2, indicating a significant change in the trend of depression scores with respect to SATI

According to the three weighted binary logistic regression models, there were significant positive correlations between SATI, VATI and depression (Table 2). After full adjustment, each one-unit increase in SATI is associated with a statistically significant 1.86-fold increase in the odds of depression occurring (Model 3: OR = 1.86, 95% CI: 1.38–2.51). For every one-unit increase in VATI, the odds of the depression occurring increase by 4.53 times after full adjustment, and this association is statistically significant (Model 3: OR = 4.53, 95% CI: 1.75–11.72). After dividing SATI, VATI into quartiles, the correlation remained statistically significant. In all models, the highest quartile (Q4) of SATI, VATI were significantly associated with depression, and there were significant trend (P for trend < 0.05) (Table 2). The findings indicated that an increase in abdominal fat was related to an increased risk of the depression occurring. The restricted cubic splines (RCS) analysis revealed a linear relationship between VATI and depression (P-overall < 0.001, P-nonlinear > 0.05), whereas SATI exhibited a nonlinear relationship with depression (P-overall < 0.001, P-nonlinear < 0.05) (Fig. 2). The RCS curve identified a critical inflection point at SATI = 0.55 kg/m^2^. This inflection point signifies a notable shift in the relationship between SATI and depression. Below this threshold, the slope was negative but not statistically significant (β = − 0.26, 95% CI = − 1.11 to 0.59, *P* = 0.555), implying that any perceived reduction in depression risk lacks statistical support. In contrast, above the 0.55 kg/m² threshold, the slope became significantly positive (β = 0.92, 95% CI = 0.54–1.30, *P* < 0.001), suggesting that the protective effect is nullified and the relationship is reversed, with each unit increase in SATI being significantly associated with an increased risk of depression.

**Table 2:** Weighted logistic regression analysis results for the associations between subcutaneous adipose tissue index and visceral adipose tissue index with depression

	Model 1		Model 2		Model 3	
	OR（95% CI)	P-value	OR（95% CI）	P-value	OR（95% CI）	P-value
Continuous variables						
**SATI**	2.81(2.19-3.6)	<0.001	2.18(1.64-2.91)	<0.001	1.86(1.38-2.51)	<0.001
**VATI**	11.49(5.28-25.02)	<0.001	11.63(4.78-28.29)	<0.001	4.53(1.75-11.72)	0.002
SATI quartiles						
**Q1**	Reference		Reference		Reference	
**Q2**	0.87(0.66-1.15)	0.338	0.83(0.62-1.11)	0.207	0.86(0.64-1.16)	0.322
**Q3**	1.19(0.91-1.56)	0.204	1.03(0.77-1.37)	0.853	0.98(0.73-1.32)	0.915
**Q4**	2.09(1.63-2.68)	<0.001	1.63(1.23-2.16)	0.001	1.45(1.08-1.95)	0.013
** P for trend**	<0.001		<0.001		0.005	
VATI quartiles						
**Q1**	Reference		Reference		Reference	
**Q2**	1.12(0.86-1.47)	0.389	1.23(0.93-1.63)	0.155	1.21(0.91-1.62)	0.181
**Q3**	1.22(0.94-1.59)	0.126	1.35(1.01-1.79)	0.041	1.19(0.89-1.58)	0.236
**Q4**	1.86(1.46-2.38)	<0.001	1.96(1.46-2.63)	<0.001	1.55(1.15-2.10)	0.004
** P for trend**	<0.001		<0.001		0.002	

Model 1: no covariates were adjusted, Pseudo R² (McFadden) = 0.016

Model 2: adjusted for age, sex, race, education. Pseudo R² (McFadden) = 0.036

Model 3: adjusted for age, sex, race, education and PIR, alcohol drinking, smoking status, high blood pressure, diabetes, physical activity. Pseudo R² (McFadden) = 0.104

*OR* Odds ratio, *CI* Confidence intervals, *SATI* Subcutaneous adipose tissue index, *VATI* Visceral adipose tissue index

### Subgroup analyses and the interaction test

Subgroup analyses were conducted using multiple stratification criteria, including demographic characteristics, personal habits, and comorbidities. Refer to Table 3 for detailed results. The significant association between SATI and the risk of depression persisted across various subgroups, including females, individuals identifying as Other Hispanic, Non-Hispanic White, or Other Race, those with education levels from 9th to 11th grade and college graduate or above, all PIR categories, never smokers, individuals with varying levels of physical activity, alcohol consumers, and those with or without hypertension and diabetes. An interaction between SATI and diabetes status was observed at the nominal level (*P* < 0.05) but did not remain significant after Bonferroni correction. The significant association between VATI and depression risk persisted across multiple subgroups, including females, those identifying as Other Hispanic, Other Race, individuals with education levels from 9th to 11th grade and college graduate or above, PIR groups (≤ 1.3 and > 3.5), non-smokers, alcohol consumers, and individuals without hypertension or diabetes. Interaction effects between VATI and sex, education level, and smoking status were observed at the nominal level (*P* < 0.05). After Bonferroni correction across nine tests (threshold *P* < 0.0056), only the VATI × Sex interaction remained significant (*P* = 0.002).

**Table 3:** Association between subcutaneous adipose tissue index and visceral adipose tissue index with depression in subgroups the associations

	**SATI**			**VATI**		
	**OR(95%CI)**	**P value**	**P interaction**	**OR(95%CI)**	**P value**	**P interaction**
Gender			0.119			**0.002**
Male	1.32(0.73-2.41)	0.363		0.29(0.04-2.34)	0.245	
Female	2.19(1.54-3.12)	<0.001		5.01(1.18-21.20)	0.029	
Race			0.812			0.942
Mexican American	0.94(0.35-2.51)	0.895		3.74(0.39-35.86)	0.252	
Other Hispanic	2.64(1.13-6.17)	0.025		28.67(2.98-276.21)	0.004	
Non-Hispanic White	1.90(1.22-2.97)	0.005		2.77(0.76-10.03)	0.121	
Non-Hispanic Black	1.31(0.81-2.14)	0.275		2.29(0.27-19.48)	0.447	
Other Race	3.4(1.55-7.44)	0.002		29.35(1.08-798.84)	0.045	
Education level			0.090			**0.027**
Less than 9th grade	1.31(0.58-2.97)	0.515		3.31(0.23-48.24)	0.381	
9-11th grade	2.19(1.16-4.14)	0.016		7.24(1.01-51.69)	0.048	
High school graduate	1.62(0.98-2.68)	0.061		2.61(0.54-12.69)	0.234	
Some college or AA degree	1.43(0.72-2.86)	0.306		3.72(0.43-32.23)	0.233	
College graduate or above	6.23(1.70-22.81)	0.006		106.79(2.84-4015.17)	0.012	
PIR			0.685			0.143
≤1.3	1.98(1.32-2.97)	0.001		10.74(2.78-41.42)	0.001	
>1.3，≤3.5	1.69(1.02-2.81)	0.041		1.13(0.23-5.70)	0.878	
>3.5	2.74(1.18-6.38)	0.019		20.63(1.94-219.29)	0.012	
Smoking status			0.497			**0.031**
Never smoking	2.32(1.5-3.59)	<0.001		32.24(7.27-142.9)	<0.001	
Former smoking	1.47(0.72-3.00)	0.295		0.50(0.06-3.93)	0.514	
Current smoking	1.42(0.85-2.35)	0.179		1.68(0.37-7.58)	0.502	
Physical Activity			0.089			0.355
Inactive	1.61(0.96-2.71)	0.069		2.81(0.28-27.9)	0.379	
Less active	3.37(1.51-7.52)	0.003		10.08(0.99-103.06)	0.051	
Active	1.63(1.06-2.50)	0.025		3.41(0.96-12.13)	0.058	
Alcohol drinking			0.620			0.277
Never	1.12(0.54-2.33)	0.759		2.00(0.39-10.41)	0.408	
Mild and moderate	2.60(1.30-5.21)	0.007		28.52(1.8-451.39)	0.017	
Heavy	1.88(1.27-2.78)	0.002		3.82(1.04-14.09)	0.044	
HBP			0.873			0.578
No	1.90(1.30-2.78)	0.001		6.13(1.70-22.08)	0.006	
Yes	1.84(1.14-2.96)	0.012		2.95(0.72-12.03)	0.131	
Diabetes			**0.035**			0.209
No	1.66(1.2-2.31)	0.002		6.13(1.70-22.08)	0.006	
Yes	4.00(1.86-8.6)	<0.001		2.95(0.72-12.03)	0.131	

All interaction P-values are Bonferroni-corrected across the nine interaction tests (significance threshold P < 0.0056). The VATI × Sex interaction remained significant (P = 0.002)

*OR* Odds ratio, *CI* Confidence intervals, *PIR* Poverty-to-income ratio, *HBP* High blood pressure, *SATI* Subcutaneous adipose tissue index, *VATI* Visceral adipose tissue index

### Mediating effects of NHR on the relationship between SATI, VATI and depression

**Fig. 3:**
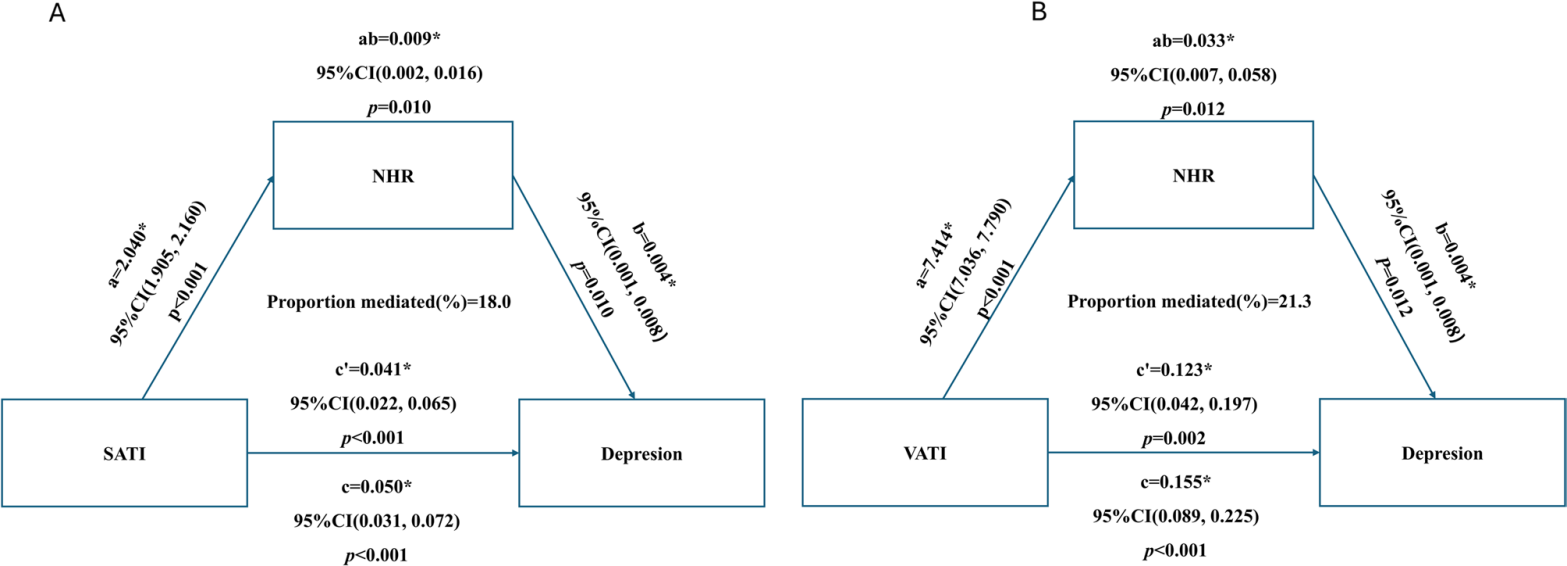
Path diagram of the mediation analysis of nutrophil-to-high-density lipoprotein cholesterol ratio(NHR) on the relationship between abdominal adipose tissue and depression. **a** reflects the path coefficient for the direct effect of SATI or VATI on NHR. **b** reflects the path coefficient for the effect of NHR on depression. ab: reflects the path coefficient for the indirect effect of SATI or VATI on depression through NHR. c′: reflects the path coefficient for the direct effect of SATI or VATI on depression, controlling for the effect of NHR. SATI-Subcutaneous Adipose Tissue Index, VATI-Visceral Adipose Tissue Index. * *P* < 0.05

**Fig. 4:**
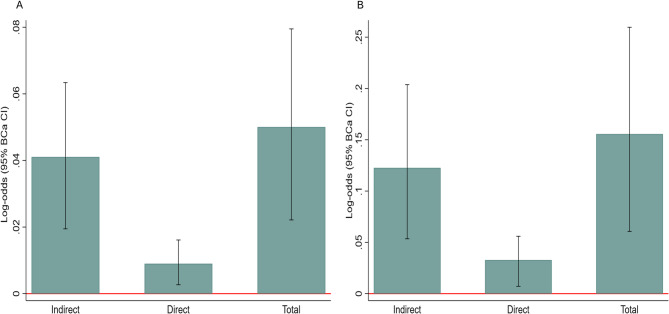
**A** The total effect, direct effect, and NHR-mediated indirect effect of SATI on depression; (**B**) The total effect, direct effect, and NHR-mediated indirect effect of VATI on depression

Mediation analyses indicated that the associations between SATI, VATI, and depression were partially mediated by NHR, as depicted in Fig. 3. Specifically, using a bias-corrected and accelerated (BCa) bootstrap method with 1,000 resamples and following comprehensive adjustment (model 3), the proportion of the indirect effect of SATI on depression mediated by NHR was 13.2% (β = 0.009, 95% BCa CI:0.002 ~ 0.016, *P* = 0.010), while for VATI, this proportion was 21.3% (β = 0.033, 95% BCa CI: 0.007 ~ 0.058, *P* = 0.012). Furthermore, both SATI and VATI demonstrated significant direct effects (for SATI, β = 0.041, 95% BCa CI: 0.022 ~ 0.065, *P* < 0.001; for VATI, β = 0.123, 95% BCa CI: 0.045 ~ 0.201, *P* = 0.002). Figure 4 provides a visual representation of the total, direct, and NHR-mediated indirect effects connecting SATI and VATI to depression.

To further investigate the potential moderating role of sex in the pathways of SATI and VATI influencing depression through NHR, we conducted 1,000-iteration BCa bootstrap mediation analyses using a fully adjusted model on a sample comprising 5,913 men and 5,654 women. The principal findings are as follows: For the pathway SATI → NHR → Depression in women, the indirect effect was 0.014 (95% BCa CI: 0.003–0.025), accounting for 26.4% (5.7%–47.2%) of the total effect, indicating significant mediation. In men, the indirect effect was 0.006 (95% BCa CI: −0.003–0.018), which includes zero, suggesting that NHR does not mediate the relationship; however, the total effect of SATI on depression remained significant (c = 0.036, 95% CI: 0.003–0.069). For the pathway VATI → NHR → Depression in women, the indirect effect was 0.047 (95% BCa CI: 0.003–0.092), accounting for 21.3% (1.4%–41.6%) of the total effect, indicating significant mediation. In men, the indirect effect was 0.032 (95% CI: 0.002–0.066), which is significant, yet the total effect spans zero (c = 0.008, 95% CI: −0.082–0.125), demonstrating a “mediation-without-effect” pattern. Tests for differences revealed that the sex difference in the percentage mediated by SATI was significant (95% BCa CI did not contain zero), whereas the sex difference for VATI was not significant (95% BCa CI contained zero). Detailed information is provided in Supplementary Table 1.

## Discussion

This study utilized weighted analysis methods to determine a weighted prevalence of depression at 8.10%, underscoring depression as a significant public health issue within the study sample. This finding is consistent with the global trend of increasing depression prevalence, particularly during the COVID-19 pandemic, which has seen a marked rise in depression incidence [25, 26]. Participants had a mean age of 38.81 years, with 47.86% being female, who showed a higher prevalence of depression than males. This sex disparity is consistent with existing literature, which often suggests that females have a higher risk of developing depression [25, 27–29]. Potential explanations for this difference include biological factors (e.g., variations in sex hormone levels), psychological factors (e.g., higher levels of neuroticism), and socio-environmental factors (e.g., sex inequality and stressors).

Our study first examined the relationship between SATI, VATI and depression. Results showed both indices are significantly positively correlated with depression. This finding is consistent with existing research, suggesting that fat distribution may have an impact on mental health [8, 9, 30]. The restricted cubic splines (RCS) analysis indicated a linear relationship between VATI and depression, suggesting that an increase in visceral fat directly heighten the risk of depression. In contrast, SATI showed a nonlinear association with depression, implying that subcutaneous fat might protect mental health up to a certain point, after which it could become detrimental. This distinction may be attributed to the differing physiological functions and metabolic characteristics of these adipose tissues [31–33]. Visceral adipose tissue is closely linked to systemic metabolism and inflammation, with its accumulation potentially leading to chronic inflammatory states that negatively impact brain function and mood regulation [10, 11]. In contrast, subcutaneous adipose tissue may influence mental health indirectly by modulating hormone levels and energy metabolism [32, 34, 35]. Furthermore, the distribution of adipose tissue may be correlated with individual lifestyle, dietary habits, and genetic factors, which collectively impact the onset and progression of depression. In a large-scale prospective study, researchers found interactions between lifestyle and factors like BMI, body fat percentage, and fat mass index regarding depression risk [36]. Existing literature indicates that dietary habits, by influencing fat distribution and metabolic health, have a significant impact on mental health [37, 38]. A Mendelian randomization (MR) study utilized genome-wide association study (GWAS) data from the UK Biobank and the Psychiatric Genomics Consortium identified obesity and short stature as causal risk factors for depression [39].

Our study’s subgroup analyses showed that SATI and VATI vary in their links to depression risk across different subgroups. SATI’s association with depression risk interacted with diabetes status, while VATI’s association interacted with sex, educational level and smoking status. This indicates that demographic and health factors may influence the link between abdominal fat distribution and depression risk. Following the application of the Bonferroni correction, the interaction between visceral adipose tissue index (VATI) and sex emerged as the only statistically significant interaction (*P* = 0.002). Stratified analyses indicated that the positive correlation between VATI and depression was evident exclusively in females, with no corresponding association identified in males. This sex-specific effect aligns with the baseline distribution: females comprised 60.54% of participants with elevated PHQ-9 scores, compared to 46.74% of those without (Table 1). Potential explanations for this disparity include sex differences in body fat distribution, hormonal influences, and psychosocial factors, all of which merit further investigation.

NHR is a superior index compared to traditional markers like CRP and IL-6, as it combines neutrophil-driven immune activation with HDL-C’s anti-inflammatory effects, providing a more comprehensive view of the adiposity-inflammation-depression axis. In cardiac-arrest survivors [40], NHR showed a higher ROC-AUC of 0.74, outperforming CRP (0.58) and slightly exceeding IL-6 (0.69), indicating better prediction of adverse outcomes. Easily calculated from routine tests, NHR is cost-effective and accessible, making it the study’s primary biomarker. Similarly, research conducted within a Taiwanese community cohort corroborated the efficacy of NHR in identifying individuals at elevated cardiovascular risk [41]. However, findings from this cohort also suggested that NHR cut-off values might be population-specific and that single-point measurements could have been influenced by acute-phase variability, underscoring the necessity for external validation. Therefore, the mediation analysis demonstrated that NHR partially mediated the relationship between the SATI, VATI and depression. This finding aligns with a study conducted on U.S. adults, which identified a positive correlation between elevated NHR levels and an increased risk of depression [19]. Specifically, that study indicated that each one-unit increase in NHR was associated with a 3% increase in the risk of depression (Odds Ratio = 1.03, 95% Confidence Interval: 1.01–1.05, *P* < 0.0005). Additionally, the relationship between NHR and depression exhibited a U-shaped pattern, with a significant breakpoint identified at an NHR value of 6.97, further underscoring the potential of NHR as a biomarker. Our study demonstrated that NHR partially mediates the relationship between abdominal fat distribution and depression, as determined through mediation analysis. This finding not only substantiates the association between NHR and depression but also elucidates the underlying mechanism, suggesting that abdominal fat may impact brain function and mood by influencing systemic inflammation and metabolic disorders. As a straightforward and easily measurable biomarker, NHR holds significant potential for widespread application. Our study offers new evidence for using NHR in depression detection and personalized therapy, highlighting its clinical value.

This study represents the inaugural effort to integrate interaction analysis with sex-stratified mediation testing to systematically elucidate the sex-specific mechanisms by which SATI and VATI influence depression through NHR. Three principal conclusions are drawn: First, SATI: discrepancy between interaction and mediation. At the population level, the interaction between SATI and sex was not statistically significant (*p* = 0.119). However, sex-stratified mediation analysis revealed a significant indirect pathway in women, accounting for 26.4% of the total effect. In contrast, NHR did not mediate the relationship in men, as the indirect effect included zero. This finding underscores the notion that “a non-significant interaction does not equate to the absence of mechanistic differences.” It is plausible that men may mitigate SATI-related risks through compensatory or counteracting mechanisms (e.g., social support, hormonal buffering), aligning the overall effect with that observed in women and thereby obscuring the interaction signal. Thus, sex differences are embedded within the pathway rather than in the overall effect magnitude. Second, VATI: notable interaction coexisting with “mediation without effect.” The interaction between VATI and sex was statistically significant (*p* = 0.002), although the sex difference in the mediated percentage was not significant, as the confidence interval included zero. In women, the pathway from VATI to NHR to depression was fully significant, indicating an amplified risk. In men, while VATI significantly increased NHR and the indirect effect was significant, the total effect was negligible, demonstrating “mediation without effect.” This suggests that the downstream threshold or pathway connecting NHR to depression is attenuated in men, negating the amplifying effect seen in women. Third, clinical and public health implications: for SATI, interventions should focus on blocking the NHR pathway in women, such as by reducing exposure or suppressing NHR elevation. For VATI, attention to NHR is warranted for both sexes, although women benefit more significantly; further investigation into downstream targets, such as inflammatory modulation or neurotransmitters, is needed for men to elucidate the “no-effect” phenomenon. Overall, integrating interaction analysis with stratified mediation effectively identifies the specific conditions, populations, and mechanisms through which sex differences manifest, thereby offering direct evidence for the development of sex-specific strategies for depression prevention.

Our study possesses several notable strengths. We employed innovative methodologies, utilizing DXA to measure SATI and VATI with precision, and discovered a significant positive correlation between these indices and depression. Our RCS revealed a linear association between VATI and depression, and a nonlinear association between SATI and depression, providing novel insights into their intricate relationship. Subgroup analyses further elucidated the interaction effects of demographic and health variables on the relationship between abdominal fat distribution and depression risk, helping to identify high-risk groups for targeted preventive interventions. Additionally, we applied mediation analysis to demonstrate that NHR partially mediates the relationship between abdominal fat and depression, suggesting that abdominal fat may influence brain function and mood through pathways involving systemic inflammation and metabolic disorders. Our findings also emphasize the importance of optimizing fat distribution through lifestyle modifications and health management to mitigate the risk of depression, which holds substantial implications for clinical practice and public health policy.

Nevertheless, several limitations are present in this study. The sample mainly consisted of U.S. adults, potentially restricting the findings’ applicability to wider populations. To enhance the applicability of future research, it is recommended to incorporate more diverse samples. As this is a cross-sectional study, it can only demonstrate associations and cannot establish causality. Future longitudinal or interventional designs are needed to clarify the direction of the relationship between fat distribution and depression, and to validate the hypothesized inflammatory and metabolic mechanisms. Additionally, although lifestyle interventions were highlighted, the study did not explore their specific components in detail. Subsequent research could focus on evaluating the effectiveness of particular strategies, such as dietary modifications or exercise programs, in optimizing fat distribution and reducing the risk of depression. Finally, the PHQ-9 is a screening instrument for depressive symptoms and not a diagnostic tool; consequently, our findings reflect the severity of depressive symptoms rather than a clinical diagnosis of major depressive disorder.

## Conclusion

Our study employed innovative methodologies to reveal a significant positive correlation between SATI and VATI with depression, with NHR partially mediating this association. Visceral fat seems to directly raise depression risk, while subcutaneous fat offers no protection below a SATI of 0.55 kg/m² and increases depression risk above this level. Subgroup analyses identified specific high-risk populations, facilitating targeted prevention strategies. These findings offer a novel perspective on the interplay between fat distribution and mental health, and suggest potential biological markers for future preventive and interventional strategies aimed at addressing depression. Optimizing fat distribution and promoting healthy lifestyle choices may mitigate the risk of depression.

## Supplementary Information

Supplementary Material 1.

## Data Availability

Publicly available datasets were analyzed in this study. These data can be found at the following URL: https://www.cdc.gov/nchs/nhanes/.
