# Supplementary material for: Neutrophil-to-high-density lipoprotein cholesterol ratio (NHR) mediates the relationship between abdominal fat index and depression in a cross-sectional study
Source: BMC Psychiatry. 2025 Oct 29;25:1035. doi: 10.1186/s12888-025-07498-5 (PMC12574252; doi:10.1186/s12888-025-07498-5)
Supplement: Supplementary file 1 — Supplementary Material 1. [file 12888_2025_7498_MOESM1_ESM.docx]

**Table** **1** Bootstrap Mediation Path Coefficients and 95% BCa Confidence Intervals(Model 3).

| **SATI** | | | | | | | | | | | | |
| --- | --- | --- | --- | --- | --- | --- | --- | --- | --- | --- | --- | --- |
|  | **Female(n=5913)** | | | | |  | | **Male(n=5654)** | | | | |
| **Path** | **Est.** | **Bias** | **SE** | **95% BCa CI** |  | | **Est.** | | | **Bias** | **SE** | **95% BCa CI** |
| a (SATI → NHR) | 1.736 | 0.0001 | 0.072 | 1.585-1.869 |  | | 2.618 | | | 0.002 | 0.113 | 2.403 -2.827 |
| b (NHR → depression) | 0.008 | 0.0001 | 0.003 | 0.002-0.015 |  | | 0.002 | | | 0.000 | 0.002 | -0.001 -0.007 |
| c (SATI → depression total) | 0.053 | 0.001 | 0.015 | 0.024-0.083 |  | | 0.036 | | | 0.000 | 0.016 | 0.003 -0.069 |
| c′ (SATI → depression direct) | 0.039 | 0.0007 | 0.016 | 0.007-0.070 |  | | 0.029 | | | 0.000 | 0.017 | -0.003 -0.064 |
| ab (indirect) | 0.014 | 0.0003 | 0.006 | 0.003-0.025 |  | | 0.006 | | | 0.000 | 0.005 | -0.003 -0.018 |
|  |  |  |  |  | |  | |  | |  |  |  |
| **VATI** | | | | | | | | | | | | |
|  | **Female(n=5913)** | | | | |  | | **Male(n=5654)** | | | | |
| **Path** | **Est.** | **Bias** | **SE** | **95% BCa CI** |  | | **Est.** | | **Bias** | | **SE** | **95% BCa CI** |
| a (VATI → NHR) | 6.892 | 0.016 | 0.257 | 6.330 -7.345 |  | | 8.411 | | 0.014 | | 0.386 | 7.619 -9.155 |
| b (NHR → depression) | 0.007 | 0.000 | 0.003 | 0.001 -0.013 |  | | 0.004 | | 0.000 | | 0.002 | 0.000 -0.008 |
| c (VATI → depression total) | 0.221 | 0.002 | 0.051 | 0.121 -0.319 |  | | 0.008 | | -0.001 | | 0.051 | -0.082 -0.125 |
| c′ (VATI → depression direct) | 0.174 | 0.000 | 0.055 | 0.063 -0.279 |  | | -0.024 | | -0.000 | | 0.053 | -0.116 -0.101 |
| ab (indirect) | 0.047 | 0.001 | 0.022 | 0.003 -0.092 |  | | 0.032 | | -0.000 | | 0.016 | 0.002 -0.066 |
| Note: SATI, subcutaneous adipose tissue index; NHR, neutrophil-to-high-density lipoprotein cholesterol ratio; VATI, visceral adipose tissue index; Est., observed estimate; SE, standard error; CI, confidence intervals; BCa, bias-corrected and accelerated bootstrap. Confidence intervals excluding zero are considered statistically significant. | | | | | | | | | | | | |
